# A Global Survey of COVID-19 Vaccine Acceptance Among Healthcare Workers

**DOI:** 10.3389/fpubh.2021.794673

**Published:** 2022-02-08

**Authors:** Mohammed Noushad, Samer Rastam, Mohammad Zakaria Nassani, Inas Shakeeb Al-Saqqaf, Mudassir Hussain, Ali Ango Yaroko, Mohammed Arshad, Abdullahi Musa Kirfi, Pradeep Koppolu, Fayez Hussain Niazi, Ali Elkandow, Mahmoud Darwish, Ahmad Salim Abdalla Nassar, Sami Osman Abuzied Mohammed, Nasser Hassan Abdalrady Hassan, Ghadah Salim Abusalim, Abdulaziz Samran, Anas B Alsalhani, Amir Mohiddin Demachkia, Renata Marques de Melo, Norhayati Luddin, Adam Husein, Adnan Habib, Firas Suleyman, Hussein Ali Osman, Mohammed Sadeg Al-Awar, Mohiddin R. Dimashkieh, Lingam Amara Swapna, Ali Barakat, Ali Alqerban

**Affiliations:** ^1^Dar Al Uloom University, Riyadh, Saudi Arabia; ^2^Vision Colleges, Riyadh, Saudi Arabia; ^3^University Sains Malaysia, Penang, Malaysia; ^4^Karachi Medical and Dental College, Karachi, Pakistan; ^5^Usman Danfodiyo University Teaching Hospital, Sokoto, Nigeria; ^6^Kannur Dental College, Kerala, India; ^7^Abubakar Tafawa Balewa University Teaching Hospital, Bauchi, Nigeria; ^8^Hamad Medical Corporation, Doha, Qatar; ^9^Ministry of Health, Amman, Jordan; ^10^King Abdullah Medical City, Makkah, Saudi Arabia; ^11^King Faisal Medical Complex, Taif, Saudi Arabia; ^12^Prince Sattam Bin Abdulaziz University, Al-Kharj, Saudi Arabia; ^13^São Paulo State University (UNESP), São José dos Campos, São Paulo, Brazil; ^14^Universiti Sains Malaysia, Kelantan, Malaysia; ^15^College of Dentistry, Aleppo University, Aleppo, Syria; ^16^University of Health Sciences, Istanbul, Turkey; ^17^Umma University, Kajiado, Kenya; ^18^Al-Razi University, Sana'a, Yemen

**Keywords:** COVID-19, vaccine acceptance, low-income countries, population immunity, vaccine inequality

## Abstract

**Objectives:**

Even though several effective vaccines are available to combat the COVID-19 pandemic, wide disparities in vaccine distribution, and vaccine acceptance rates between high- and low-income countries appear to be major threats toward achieving population immunity. Our global descriptive study aims to inform policymakers on factors affecting COVID-19 vaccine acceptance among healthcare workers (HCWs) in 12 countries, based on income index. We also looked for possible predictors of vaccine acceptance among the study sample.

**Methods:**

A structured questionnaire prepared after consultation with experts in the field and guided by the “Report of the SAGE working group on vaccine hesitancy” was administered among 2,953 HCWs. Upon obtaining informed consent, apart from demographic information, we collected information on trust in vaccines and health authorities, and agreement to accept a COVID-19 vaccine.

**Results:**

Although 69% of the participants agreed to accept a vaccine, there was high heterogeneity in agreement between HCWs in low and lower-middle income countries (L-LMICs) and upper-middle- and high-income countries (UM-HICs), with acceptance rates of 62 and 75%, respectively. Potential predictors of vaccine acceptance included being male, 50 years of age or older, resident of an UM-HIC, updating self about COVID-19 vaccines, greater disease severity perception, greater anxiety of contracting COVID-19 and concern about side effects of vaccines.

**Conclusions:**

COVID-19 vaccine acceptance among HCWs in L-LMICs was considerably low as compared to those from UM-HICs. The lowest vaccine acceptance rates were among HCWs from the African continent. This underlines the need for the implementation of country-specific vaccine promotion strategies, with special focus on increasing vaccine supply in L-LMICs.

## Introduction

The SARS-CoV-2 that causes COVID-19 has been responsible for millions of deaths worldwide. Although preventive measures have been able to limit mortality and morbidity, any laxity therein, or the introduction of new mutations have usually been shown to cause recurring waves of infections, many a times even more destructive than the first one (1). As of Sepember 2021, at least eight notable mutations including the highly transmissible Delta variant has been identified, causing global concern, and forcing countries to impose repeated border restrictions (2). Therefore, unlike endemic diseases, pandemics being global in nature require collective global action to translate to global population immunity.

Two pathways exist that could lead to population immunity, either through previous infection or through immunization. According to the World Health Organisation (WHO), immunization programs prevent an estimated 2–3 million deaths every year globally (3). Since aiming for population immunity to COVID-19 through previous infection could lead to excess mortality and morbidity, and a huge socio-economic burden, especially as a consequence of the emerging mutations, the ideal strategy would be to attain it through mass immunization programs (1, 4). However, two major hurdles exist: vaccine hesitancy, *a delay in acceptance, or refusal of vaccination despite availability of vaccination services*, and vaccine inequality or lack of access to vaccines, a trend that has crippled mostly low and lower middle-income nations (L-LMICs) (5). As of 30 September 2021, <1% of people in low-income countries (LICs), and just 10% in lower-middle-income countries (LMICs) are fully vaccinated, compared with more than half in high-income countries (HICs) (6). Therefore, attaining global population immunity necessitates a two-pronged approach; combating vaccine hesitancy and promoting vaccine equality.

Although vaccine inequality is a complex issue that must be dealt with by policymakers and stakeholders, vaccine acceptance is mostly based on individual preferences. It is context, time and geographic location specific, and could be due to complacency around the disease, availability, ease of access and safety concern of vaccines, etc. (7). Since attitudes toward vaccines could be multifactorial, determining the predictors of vaccine acceptance could guide policymakers in formulating appropriate healthcare strategies for targeting vulnerable populations. This becomes all the more important in light of the anti-vaccine movements and protests across several countries, sometimes even supported by healthcare workers (HCWs) (8, 9).

Although, most of the focus in L-LMICs has been on vaccine inequality, lack of access and infrastructure, inefficient cold storage facilities, etc., information on vaccine acceptance, especially among HCWs is lacking. Our study aims to fill this gap by comparing vaccine acceptance in a sample of six L-LMICs and six upper middle income and high-income countries (UM-HICs) with wide geographic coverage, spanning over Asia, Africa, Europe, and South America.

Since HCWs form the frontline combatants against disease outbreaks, and are one of the most vulnerable groups, they have rightly been prioritized to receive vaccination against COVID-19. As of 24 May 2021, the WHO has estimated 115,000 HCWs to have died due to COVID-19 (10). Nonetheless, studies have shown wide variations of vaccine acceptance among HCWs, from as high as 95 percent to as low as 28 percent (11, 12). Since HCWs could act as messengers to the general public in vaccine promotion, we found it appropriate to determine the predictors of vaccine acceptance among them. Our study aimed to explore intention to accept COVID-19 vaccines among HCWs and to identify potential predictors of vaccine acceptance among them in 12 countries grouped on the basis of income index.

## Materials and Methods

### Study Design

A cross-sectional online self-administered survey was conducted among HCWs in 12 countries (Sudan, Pakistan, Nigeria, Kenya, India, Egypt, Turkey, Qatar, Malaysia, Saudi Arabia, Jordan, and Brazil) from February to the middle of April 2021. We grouped the countries into six L-LMICs and six UM-HICs (13). The ‘Report of the SAGE working group on vaccine hesitancy' and opinions from experts in the field were used in preparing the questionnaire (14). A pilot study was initially carried out on 10 participants, after which expert opinion was taken from four specialists in the field. The survey questionnaire developed on Google Forms was distributed online. The questionnaire required <5 min to complete. Participation was voluntary and the participants provided informed consent on the survey platform before proceeding to the survey items. The participants' anonymity was guaranteed during the data collection process. Participants were reminded only once, on failure to complete the survey form.

This study was approved by the Research Committee of College of Dentistry, Dar Al Uloom University, Saudi Arabia (COD/IRB/2020/2).

### Sample

Participants (*n* = 2,963) included HCWs from various specialties from twelve countries. HCWs expected to come in contact with patients were only included in the study. Students who had entered the clinical training level in their fields of study were also included in the study. Other students in the healthcare field were excluded from the study. Participants below the age of 18 years were not included in the study. Participants were not compensated for participation in the study. The survey form was designed in such a way that only completed forms would qualify for submission.

### Measures

#### Trust in Vaccines, Vaccine Manufacturers, and Health Authorities

General attitudes toward vaccines were measured using a set of 6 items. First, participants were asked if vaccines were really necessary to overcome the pandemic. Assessment questions on perceived trust in vaccine manufacturers included, whether participants trusted vaccines of only specific companies, whether vaccine manufacturers followed recommended development and production guidelines, concerns of commercial profiteering, risk of side effects of vaccines and whether manufacturers were open about disclosing the side effects of vaccines. Responses were rated on a five-point scale from 1 “strongly agree” to 5 “strongly disagree.” Participants' attitudes toward health care system were measured using a set of 2 items. Participants were asked if they were happy with the health authorities' handling of the pandemic, and their management of vaccination campaigns. Responses were rated on a five-point scale from 1 “strongly agree” to 5 “strongly disagree.”

#### Intention to Vaccinate

The vaccine intention was measured using a set of 7 items. First, participants were asked if vaccines should be made mandatory and if the participant intended to get vaccinated. Further questions included fear of the vaccine, care for others who would be in greater need for the vaccine, intention to protect others with weaker immunity, willingness/unwillingness to take the vaccine if required to pay for it, and fear of side effects from second dose. Responses were rated on a six-point scale from 1 “strongly agree” to 5 “strongly disagree.”

#### Exploratory Variables

Socio-demographic factors included age group, sex, nationality, region of current work/study place, type of work/study (governmental/private/both), and profession. Participants' report on chronic medical condition (e.g., asthma, diabetes, hypertension, heart disease, and/or cancer) was used to indicate the presence or absence of pre-existing co-morbidity. Other variables included, participants' self-updating on COVID-19 vaccine development, prior infection with COVID-19, perception of COVID-19 severity, compliance with government COVID-19 guidelines and anxiety toward contracting COVID-19.

### Statistical Analysis

The main outcome of this study was intention to vaccinate. Sample statistics for each country and for the totals of low- and high-income countries were expressed as numbers and percentages for different socio-economical, health and COVID-19 vaccination parameters (Table 1). Details of responses for all questionnaire items among all countries were expressed as number and percentages (Table 2).

**Table 1:** Sample characteristics: Data are presented as *n* (%).

**Sample size**	**L-LMICs**							**UM-HICs**							**Total**
	**Egypt**	**India**	**Kenya**	**Nigeria**	**Pakistan**	**Sudan**	**Total**	**Brazil**	**Jordan**	**KSA**	**Malaysia**	**Qatar**	**Turkey**	**Total**	
**Total**	151	282	102	206	453	143	**1,337**	223	131	674	222	148	228	1,626	**2,963**
**Sex**															
Male	85 (56)	73 (26)	32 (31)	141 (68)	64 (14)	63 (44)	**458 (34)**	61 (27)	57 (44)	350 (52)	69 (31)	104 (70)	86 (38)	**727 (45)**	**1,185 (40)**
Female	66 (44)	209 (74)	70 (69)	65 (32)	389 (86)	80 (56)	**879 (66)**	162 (73)	74 (56)	324 (48)	153 (69)	44 (30)	142 (62)	**899 (55)**	**1,778 (60)**
**Age**															
18–29 years	45 (30)	183 (65)	42 (41)	104 (50)	425 (94)	39 (27)	**838 (63)**	145 (65)	47 (36)	392 (58)	133 (60)	35 (24)	143 (63)	**895 (55)**	**1,733 (58)**
30–49 years	88 (58)	85 (30)	52 (51)	90 (44)	23 (5)	99 (69)	**437 (33)**	54 (24)	62 (47)	251 (37)	81 (36)	98 (66)	78 (34)	**624 (38)**	**1,061 (36)**
≥ 50 years	18 (12)	14 (5)	8 (8)	12 (6)	5 (1)	5 (3)	**62 (5)**	24 (11)	22 (17)	31 (5)	8 (4)	15 (10)	7 (3)	**107 (7)**	**169 (6)**
**Nationality**															
Native	146 (97)	279 (99)	102 (100)	206 (100)	451 (100)	142 (99)	**1,326 (99)**	218 (98)	116 (89)	410 (61)	219 (99)	15 (10)	202 (89)	**1,180 (73)**	**2,506 (85)**
Foreigner	5 (3)	3 (1)	0 (0)	0 (0)	2 (0)	1 (1)	**11 (1)**	5 (2)	15 (11)	264 (39)	3 (1)	133 (90)	26 (11)	**446 (27)**	**457 (15)**
**Place of work**															
Public	67 (44)	20 (7)	61 (60)	188 (91)	380 (84)	72 (50)	**788 (59)**	77 (35)	81 (62)	154 (23)	195 (88)	128 (86)	166 (73)	**801 (49)**	**1,589 (54)**
Private	48 (32)	251 (89)	29 (28)	9 (4)	61 (13)	30 (21)	**428 (32)**	114 (51)	44 (34)	488 (72)	21 (9)	20 (14)	45 (20)	**732 (45)**	**1,160 (39)**
Both	36 (24)	11 (4)	12 (12)	9 (4)	12 (3)	41 (29)	**121 (9)**	32 (14)	6 (5)	32 (5)	6 (3)	0 (0)	17 (7)	**93 (6)**	**214 (7)**
**Comorbidity**															
No	120 (79)	245 (87)	91 (89)	172 (83)	434 (96)	115 (80)	**1,177 (88)**	182 (82)	112 (85)	573 (85)	205 (92)	123 (83)	211 (93)	**1,406 (86)**	**2,583 (87)**
Yes	31 (21)	37 (13)	11 (11)	4 (17)	19 (4)	28 (20)	**160 (12)**	41 (18)	19 (15)	101 (15)	7 (8)	25 (17)	17 (7)	**220 (14)**	**380 (13)**
**Previous COVID-19 infection**															
No	109 (72)	245 (87)	85 (83)	165 (80)	397 (88)	89 (62)	**1,090 (82)**	174 (78)	87 (66)	572 (85)	219 (99)	112 (76)	177 (78)	**1,341 (83)**	**2,431 (82)**
Yes	42 (28)	37 (13)	17 (17)	41 (20)	56 (12)	54 (38)	**247 (18)**	49 (22)	44 (34)	102 (15)	3 (1)	36 (24)	50 (22)	**284 (17)**	**531 (18)**
**Vaccinated against COVID-19**															
No	142 (9	187 (66)	81 (79)	181 (88)	368 (81)	122 (85)	**1,081 (81)**	95 (43)	106 (81)	607 (90)	113 (51)	58 (39)	130 (57)	**1,109 (68)**	**2,190 (74)**
Yes	4) 9 (6)	95 (34)	21 (21)	25 (12)	85 (19)	21 (15)	**256 (19)**	128 (57)	25 (19)	67 (10)	109 (49)	90 (61)	97 (43)	**516 (32)**	**772 (26)**

**Table 2:** Agreement to different survey questions—Data are presented as % (*n*): L-LMICs.

**Egypt**	**India**	**Kenya**	**Nigeria**	**Pakistan**	**Sudan**	**Total (low-income countries)**
**Trust in Vaccines**						
**Vaccines are necessary to overcome the COVID-19 pandemic and get back to normal life**						
48% (72)	72% (203)	55% (56)	73% (150)	76% (343)	73% (104)	69% (928)
**I trust COVID-19 vaccines of ONLY certain companies**						
39% (59)	55% (156)	34% (35)	38% (78)	56% (253)	42% (60)	48% (641)
**Vaccines against COVID-19 have been produced in a hurry without following recommended clinical trials and approval guidelines**						
70% (106)	39% (109)	50% (51)	69% (142)	36% (161)	43% (62)	47% (631)
**Companies involved in the development of the COVID-19 vaccines are doing it to make money**						
62% (94)	18% (50)	33% (34)	42% (87)	18% (81)	37% (53)	30% (399)
**COVID-19 vaccines may have side effects which may show immediately or later on in life**						
73% (110)	50% (140)	66% (67)	78% (160)	47% (214)	62% (88)	58% (779)
**Companies producing COVID-19 vaccines are open about disclosing information on the side effects of the vaccine**						
28% (42)	37% (103)	36% (37)	23% (48)	33% (151)	48% (69)	34% (450)
**Trust in Authorities**						
**The health authorities have been efficient in managing the COVID-19 pandemic so far**						
30% (45)	78% (221)	41% (42)	57% (117)	56% (255)	26% (37)	54% (717)
**The health authorities have been efficient in organizing the COVID-19 vaccination campaigns**						
27% (41)	77% (218)	42% (43)	38% (78)	69% (312)	37% (53)	56% (745)
**Intention to vaccinate**						
**I support a mandatory vaccination program for COVID-19**						
36% (55)	74% (208)	33% (34)	22% (45)	80% (362)	55% (78)	58% (782)
**I will get vaccinated with the COVID-19 vaccine**						
40% (61)	76% (215)	49% (50)	50% (104)	69% (314)	57% (81)	62% (825)
**I will wait for other people to take the COVID-19 vaccine, as I am afraid to take it myself**						
42% (64)	28% (79)	43% (44)	36% (75)	39% (175)	21% (30)	35% (467)
**I will delay taking the COVID-19 vaccine, as I feel there are others who deserve it more than me**						
54% (81)	41% (117)	27% (28)	54% (112)	56% (255)	45% (65)	49% (658)
**Getting myself vaccinated for COVID-19 is important because I can also protect people with a weaker immune system**						
62% (93)	85% (240)	61% (62)	73% (150)	79% (359)	81% (116)	76% (1,020)
**I will take the COVID-19 vaccine only if it is free**						
22% (31)	28% (68)	26% (23)	26% (48)	30% (120)	34% (44)	28% (334)
**Compared to the first dose of the COVID-19 vaccine, I fear that the second dose may have more chances to induce adverse side effects**						
39% (57)	33% (87)	38% (38)	49% (91)	31% (126)	28% (36)	35% (435)
**B) UM-HICs**							
**Brazil**	**Jordan**	**KSA**	**Malaysia**	**Qatar**	**Turkey**	**Total**	**All sample**
**Trust in Vaccines**							
**Vaccines are necessary to overcome the COVID-19 pandemic and get back to normal life**							
97% (216)	61% (80)	73% (494)	97% (215)	80% (119)	75% (171)	80% (1,295)	75% (2,223)
**I trust COVID-19 vaccines of ONLY certain companies**							
24% (54)	31% (41)	43% (290)	42% (94)	55% (82)	40% (91)	40% (652)	44% (1,293)
**Vaccines against COVID-19 have been produced in a hurry without following recommended clinical trials and approval guidelines**							
15% (34)	49% (64)	34% (230)	14% (30)	49% (73)	38% (86)	32% (517)	39% (1,148)
**Companies involved in the development of the COVID-19 vaccines are doing it to make money**							
15% (33)	54% (71)	38% (257)	18% (41)	43% (64)	40% (91)	34% (557)	32% (956)
**COVID-19 vaccines may have side effects which may show immediately or later on in life**							
25% (56)	52% (68)	45% (302)	45% (100)	49% (72)	54% (124)	44% (722)	51% (1,501)
**Companies producing COVID-19 vaccines are open about disclosing information on the side effects of the vaccine**							
61% (135)	41% (54)	41% (274)	54% (119)	39% (58)	25% (56)	43% (696)	39% (1,146)
**Brazil**	**Jordan**	**KSA**	**Malaysia**	**Qatar**	**Turkey**	**Total**	**All sample**
**Trust in Authorities**							
**The health authorities have been efficient in managing the COVID-19 pandemic so far**							
6% (13)	28% (37)	92% (620)	77% (172)	87% (129)	52% (117)	67% (1,088)	61% (1,805)
**The health authorities have been efficient in organizing the COVID-19 vaccination campaigns**							
10% (23)	45% (59)	92% (618)	87% (193)	84% (124)	57% (129)	70% (1,146)	64% (1,891)
**Intention to vaccinate**							
**I support a mandatory vaccination program for COVID-19**							
80% (179)	53% (70)	59% (396)	92% (204)	66% (98)	53% (119)	66% (1,066)	62% (1,848)
**I will get vaccinated with the COVID-19 vaccine**							
97% (216)	53% (69)	64% (431)	98% (217)	82% (121)	70% (159)	75% (1,213)	69% (2,038)
**I will wait for other people to take the COVID-19 vaccine, as I am afraid to take it myself**							
1% (3)	27% (36)	29% (194)	6% (13)	19% (28)	15% (35)	19% (309)	26% (776)
**I will delay taking the COVID-19 vaccine, as I feel there are others who deserve it more than me**							
11% (24)	50% (65)	54% (367)	16% (35)	32% (47)	29% (66)	37% (604)	43% (1,262)
**Getting myself vaccinated for COVID-19 is important because I can also protect people with a weaker immune system**							
86% (191)	66% (86)	78% (526)	96% (214)	82% (122)	71% (160)	80% (1,299)	78% (2,319)
**I will take the COVID-19 vaccine only if it is free**							
32% (36)	39% (42)	33% (210)	29% (43)	37% (37)	36% (55)	34% (423)	31% (757)
**Compared to the first dose of the COVID-19 vaccine, I fear that the second dose may have more chances to induce adverse side effects**							
11% (15)	40% (45)	30% (190)	37% (55)	58% (63)	24% (38)	31% (406)	33% (841)

Bivariate statistical analysis of the relationship between the income of the countries and various questionnaire items were conducted using Chi-squared test for trend for ordinal factors, and the Chi-squared test for categorical variables (Table 3).

**Table 3:** Comparison of responses to different survey questions between L-LMICs and UM-HICs– Data are presented as % (*n*).

**L-LMICs**	**UM-HICs**	**Total**	*p* ^*^
**Trust in Vaccines**			
**Vaccines are necessary to overcome the COVID-19 pandemic and get back to normal life**			
69.4% (928/1,337)	79.6% (1,295/1,626)	75.0% (2,223/2,963)	<0.001
**I trust COVID-19 vaccines of ONLY certain companies**			
47.9% (641/1,337)	40.1% (652/1,626)	43.6% (1,293/2,963)	<0.001
**Vaccines against COVID-19 have been produced in a hurry without following recommended clinical trials and approval guidelines**			
47.2% (631/1,337)	31.8% (517/1,626)	38.7% (1,148/2,963)	<0.001
**The companies involved in the development of the COVID-19 vaccines are doing it to make money**			
29.8% (399/1,337)	34.3% (557/1,626)	32.3% (956/2,963)	0.01
**COVID-19 vaccines may have side effects which may show immediately or later on in life**			
58.3% (779/1,337)	44.4% (722/1,626)	50.7% (1,501/2,963)	<0.001
**Companies producing COVID-19 vaccines are open about disclosing information on the side effects of the vaccine**			
33.7% (450/1,337)	42.8% (696/1,626)	38.7% (1,146/2,963)	<0.001
**Trust in Authorities**			
**The health authorities have been efficient in managing the COVID-19 pandemic so far**			
53.6% (717/1,337)	67.0% (1,088/1,624)	61.0% (1,805/2,961)	<0.001
**The health authorities have been efficient in organizing the COVID-19 vaccination campaigns**			
55.7% (745/1,337)	70.5% (1,146/1,626)	63.8% (1,891/2,963)	<0.001
**Intention to vaccinate**			
**I support a mandatory vaccination program for COVID-19**.			
58.5% (782/1,337)	65.6% (1,066/1,624)	62.4% (1,848/2,961)	<0.001
**I will get vaccinated with the COVID-19 vaccine**			
61.7% (825/1,337)	74.6% (1,213/1,625)	68.8% (2,038/2,962)	<0.001
**I will wait for other people to take the COVID-19 vaccine, as I am afraid to take it myself**			
34.9% (467/1,337)	19.0% (309/1,626)	26.2% (776/2,963)	<0.001
**I will delay taking the COVID-19 vaccine, as I feel there are others who deserve it more than me**			
49.2% (658/1,337)	37.1% (604/1,626)	42.6% (1,262/2,963)	
**Getting myself vaccinated for COVID-19 is important because I can also protect people with a weaker immune system**			
76.3% (1,020/1,337)	80.1% (1,299/1,621)	78.4% (2,319/2,958)	0.01
**I will take the COVID-19 vaccine only if it is free**			
28.3% (334/1,179)	33.9% (423/1,246)	31.2% (757/2,425)	0.003
**Compared to the first dose of the COVID-19 vaccine, I fear that the second dose may have more chances to induce adverse side effects**			
35.3% (435/1,234)	31.3% (406/1,296)	33.2% (841/2,530)	0.03

* *p was calculated based on chi-square test*.

**Figure 1:**
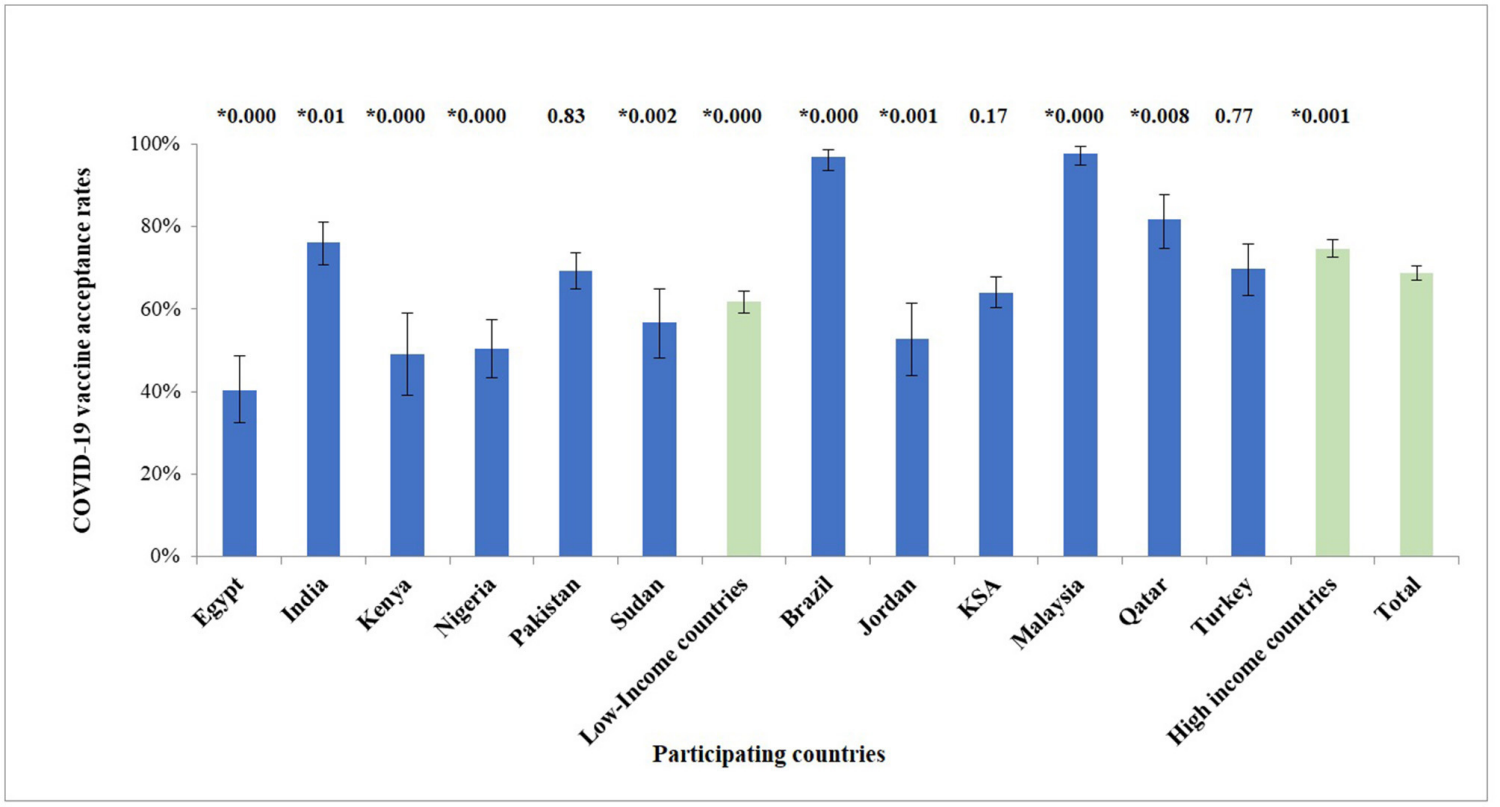
Agreement to accept a COVID-19 vaccine (proportion ± 95% confidence interval) among HCWs in different countries. Numbers above each bar represent the *p*-value for the differences between the intention to vaccinate in each country compared to the total of all the countries (0.000 means *p* < 0.001). *Denotes significant difference (*p* < 0.05).

In this study we considered the participant as willing to accept a COVID-19 vaccine if he/she agreed or strongly agreed on the item “I will get vaccinated with the Covid-19 vaccine,” or if he/she had already taken the vaccine. Intention to vaccinate and its 95% confidence interval for each country are presented in Figure 1.

Bivariate statistical analyses were conducted between “intention to vaccinate” and possible demographical, health and behavioral factors, and also between L-LMICs and UM-HICs by using Chi-squared test for trend for ordinal factors, and the Chi-squared test for categorical variables (Tables 4, 5).

**Table 4:** Agreement among HCWs in different countries to accept a COVID-19 vaccine - Data are presented as %.

**Total**	**L-LMICs**						**UM-HICs**						**Total**
	**Egypt**	**India**	**Kenya**	**Nigeria**	**Pakistan**	**Sudan**	**Brazil**	**Jordan**	**KSA**	**Malaysia**	**Qatar**	**Turkey**	
	40%	76%	49%	50%	69%	57%	97%	53%	64%	98%	82%	70%	69%
**Sex**													
Male	47%	82%	59%	49%	78%	78%	98%	65%	68%	99%	85%	83%	72%
Female	31%	74%	44%	54%	68%^**^	40%^**^	96%	43%^**^	60%^**^	97%	75%	62%^**^	67%^**^
**Age**													
18–29 years	42%	72%	45%	37%	68%	36%	97%	49%	62%	99%	54%	59%	66%
30–49 years	39%	84%	46%	66%	91%	64%	96%	50%	66%	96%	90%	87%	71%
≥50 years	44%	93%^*^	88%	58%^*^	60%	80%^*^	100%	68%	84%^*^	100%	93%^*^	86%^*^	80%^*^
**Nationality**													
Native	40%	76%	49%	51%	70%	57%	97%	55%	67%	98%	67%	69%	69%
Foreigner	60%	67%	0%	0%	0%^**^	0%	100%	33%	60%	100%	84%	73%	67%
**Place of work**													
Public	31%	80%	38%	49%	69%	64%	96%	51%	72%	97%	85%	69%	69%
Private	46%	76%	69%	56%	74%	50%	97%	61%	61%	100%	60%	76%	69%
Both	50%	73%	58%^**^	78%	67%	49%	100%	17%	66%	100%	0%^**^	65%	65%
**Comorbidity**													
No	38%	77%	49%	49%	70%	57%	97%	51%	64%	98%	81%	69%	69%
Yes	48%	73%	45%	56%	58%	57%	95%	63%	67%	100%	84%	82%	69%
**Previous COVID-19 infection**													
No	40%	76%	46%	48%	70%	62%	97%	53%	65%	98%	85%	70%	70%
Yes	40%	76%	65%	59%	66%	48%	98%	52%	56%	100%	72%	68%	63%^**^
**Updating self on the development of COVID-19 vaccines**													
No	29%	59%	47%	21%	50%	32%	94%	27%	50%	95%	36%	58%	49%
Yes	43%	79%^**^	49%	64%^**^	75%^**^	63%^**^	97%	58%^**^	67%^**^	98%	87%^**^	84%^**^	74%^**^
**Opinion about COVID-19 severity**													
Mild	25%	67%	20%	45%	67%	75%	100%	13%	44%	100%	67%	29%	49%
Moderate	32%	74%	42%	52%	63%	50%	91%	53%	59%	100%	75%	63%	62%
Severs	48%	80%^*^	57%^*^	59%	73%	58%	97%	57%	70%^*^	97%	88%^*^	76%^*^	76%^*^
**Compliance with COVID-19 preventive guidelines**													
Good	40%	85%	46%	50%	72%	60%	98%	58%	67%	98%	84%	73%	73%
Moderate	43%	70%	47%	54%	67%	50%	94%	36%	59%	98%	74%	60%	64%
Poor	33%	40%^*^	100%	40%	74%	69%	100%	75%	43%^*^	100%	0%	75%	60%^*^
**Anxiety about getting infection with COVID-19**													
Low	17%	72%	23%	28%	54%	42%	100%	38%	55%	100%	67%	63%	54%
Moderate	40%	75%	50%	60%	69%	46%	96%	49%	66%	99%	88%	70%	68%
High	50%^*^	80%	55%	58%^*^	75%^*^	78%^*^	97%	76%^*^	69%^*^	96%	80%	73%	78%^*^
**Concerned about the side effects of the vaccines**													
Yes	35%	70%	39%	51%	57%	45%	93%	41%	51%	96%	74%	60%	57%
No	54%^**^	82%^**^	69%^**^	50%	81%^**^	75%^**^	98%^**^	65%^**^	75%^**^	99%	89%^**^	81%^**^	80%^*^

* *Significance (p < 0.05) according to chi-square test for trend.*

** *Significance (p < 0.05) according to chi-square test.*

*All comparison was between the different levels of each cell*.

**Table 5:** Agreement among HCWs in L-LMICs and UM-HICs to accept a COVID-19 vaccine.

	**L-LMICs**	**UM-HICs**	**Total**	**P between high & low income**
Total	61.7% (825/1,337)	74.6% (1,213/1,625)	68.8% (2,038/2,962)	<0.001^**^
**Gender**				
Male	62.7% (287/458)	77.1% (560/726)	71.5% (847/1,184)	<0.001^**^
Female	61.2% (538/879)	72.6% (653/899)^**^	67% (1,191/1,778)^**^	<0.001^**^
**Age**				
18–29 years	61% (511/833)	71.4% (639/895)	66.4% (1,150/1,733)	<0.001^**^
30–49 years	62.2% (272/437)	77.2% (481/623)	71% (753/1,060)	<0.001^**^
≥50 years	67.7% (42/62)	86.9% (93/107)^*^	79.9% (135/169)^*^	0.003^**^
**Nationality**				
Native	61.8% (820/1,326)	77.3% (912/1,180)	69.1% (1,732/2,506)	<0.001^**^
Foreigner	45.5% (5/11)	67.6% (301/445)^**^	67.1% (306/456)	0.12
**Place of work**				
Public	58.2% (459/788)	79.8% (639/801)	69.1% (1,098/1,589)	<0.001^**^
Private	69.6% (298/428)	68.8% (503/731)	69.1% (801/1,159)	0.77
Both	56.2% (68/121)	76.3% (71/93)^**^	65.% (139/214)	0.002^**^
**Comorbidity**				
No	62.2% (732/1,177)	74.2% (1,043/1,406)	68.7% (1,775/2,583)	<0.001^**^
Yes	58.1% (93/160)	77.6% (170/219)	69.4% (263/379)	<0.001^**^
**Previous COVID-19 infection**				
No	62.6% (682/1,090)	76.2% (1,021/1,340)	70.1% (1,703/2,430)	<0.001^**^
Yes	57.9% (143/247)	67.3% (191/284)^**^	62.9% (334/531)^**^	0.026^**^
**Updating self on the development of COVID-19 vaccines**				
No	40.9% (117/286)	56.1% (174/310)	48.8% (291/596)	<0.001^**^
Yes	67.4% (708/1,051)^**^	79% (1,038/1,314)^**^	73.8% (1,746/2,365)^**^	<0.001^**^
**Opinion about COVID-19 severity**				
Mild	48.3% (57/118)	50% (28/56)	48.9% (85/174)	0.83
Moderate	57.6% (300/521)	65% (379/583)	61.5% (679/1,104)	0.011^**^
Severs	67% (468/698)^*^	81.7% (806/986)^*^	75.7% (1,274/1,684)^*^	<0.001^**^
**Compliance with COVID-19 preventive guidelines**				
Good	64.3% (366/569)	77% (890/1,156)	72.8% (1,256/1,725)	0.057
Moderate	60.4% (412/682)	68.5% (293/428)	63.5% (705/1,110)	0.007^**^
Poor	54.7% (47/86)^*^	72.5% (29/40)^*^	60.3% (76/126)^*^	<0.001^**^
**Anxiety about getting infection with COVID-19**				
Low	42.9% (85/198)	60.8% (178/293)	53.6% (263/491)	<0.001^**^
Moderate	61.9% (442/714)	73.7% (573/778)	68.% (1,015/1,492)	<0.001^**^
High	70.1% (298/425)^*^	83.4% (462/554)^*^	77.6% (760/979)^*^	<0.001^**^
**Concerned about the side effects of the vaccines**				
Yes	52% (405/779)	63.4% (457/721)	57.5% (862/1,500)	<0.001^**^
No	75.3% (420/558)^**^	83.6% (756/904)^**^	80.4% (1,176/1,426)^**^	<0.001^**^

*Data are presented as % (n).*

*All comparisons were conducted between different levels of predictors at each cell.*

* *p < 0.05 based on chi-square test for trend;*

** *p < 0.05 based on chi-square test*.

A multivariate binary logistic regression model was used to determine the predictors of vaccine acceptance. Age groups, sex, nationality, presence of any medical condition, previous infection with COVID-19, following updates on the development of vaccines against COVID-19, opinion about the severity of COVID-19, compliance with COVID-19 preventive guidelines, concern about side effects of COVID-19 vaccines and anxiety about contracting COVID-19 were included in the model and then step wisely eliminated using Wald statistic. Odds ratio and their 95% confidence interval (95% CI) are presented in Table 6.

**Table 6:** Predictors of agreement to accept a COVID-19 vaccine.

	**Odds ratio (95% odds ratio)**	***p***
**Gender**		
Male	Ref	
Female	0.75 (0.62–0.91)^*^	0.004
**Age**		
18–29 years	Ref	
30–49 years	1.27 (1.04–1.55)^*^	0.02
≥ 50 years	2.17 (1.38–3.41)^*^	0.001
**Nationality**		
Native	Ref	
Foreigner	0.53 (0.4–0.7)^*^	<0.001
**Place of work**		
Public	Ref	
Private	0.79 (0.65–0.95)^*^	0.01
Both	0.69 (0.49–0.97)^*^	0.03
**Income of the country**		
Low-income	Ref	
Mid or high income	2.08 (1.7–2.54)^*^	<0.001
**Updating self on the development of COVID-19 vaccines**		
No	Ref	
Yes	2.7 (2.2–3.31)^*^	<0.001
**Opinion about COVID-19 severity**		
Mild	Ref	
Moderate	1.17 (0.81–1.69)	0.42
Severe	1.76 (1.2–2.57)^*^	0.004
**Anxiety about getting infection with COVID-19**		
Low	Ref	
Moderate	1.74 (1.37–2.21)^*^	<0.001
High	2.57 (1.95–3.38)^*^	<0.001
**Concerned about the side effects of the vaccines**		
Yes	Ref	
No	3.06 (2.56–3.66)^*^	<0.001

* *Significance (p) is < 0.05*.

All statistical analyses were performed using IBM SPSS Statistics version 25.0 (IBM Corp. Released 2017. IBM SPSS Statistics for Windows, Version 25.0. IBM Corp, Armonk, NY, USA). The significance level was set at *p* < 0.05.

## Results

Of the 2,963 HCWs from 12 countries that participated in the current survey, 1,337 HCWs were from six L-LMICs, and 1,626 from six UM-HICs. Table 1 shows the demographics and characteristics of the study sample. Table 2 illustrates the country-wise proportions of HCWs who agreed to various survey questions. It can be noted that the majority of the study population (75%) agreed that vaccines are necessary to overcome the COVID-19 pandemic and get back to normal life. However, distribution of the agreement was wide ranging, from 48% in Egypt to 97% in Brazil and Malaysia. Of the Nigerian HCWs, 78% believed that COVID-19 vaccines may have side effects. This figure decreased tremendously to 25% among HCWs from Brazil. Overall, almost half of the HCWs (51%) believed that COVID-19 vaccines may have immediate or delayed side effects. Trust of HCWs in health authorities and their management of the COVID-19 pandemic varied among countries, from as high as 92% in Saudi Arabia to as low as 6% in Brazil.

Agreement to accept the COVID-19 vaccine was apparent among the majority of the study population (69%). While, HCWs from Brazil and Malaysia indicated the highest figures for vaccine acceptance (97, 98% respectively), those from Egypt showed the lowest (40%) (Table 2, Figure 1). Association between survey questions and income of the country indicated clear impact for country income classification on the trust in COVID-19 vaccines, trust in health authorities and vaccine acceptance (Table 3). Vaccine acceptance and trust in health authorities were significantly higher among HCWs from UM-HICs (*p* < 0.001). On the other hand, concern about side effects from COVID-19 vaccines was significantly higher among HCWs from L-LMICs (*p* < 0.001) (Table 3). Table 4 demonstrates COVID-19 vaccine intention among HCWs according to country and demographics. It is noteworthy that vaccine acceptance was higher among male HCWs across all the surveyed countries. Moreover, older HCWs had significantly greater intention to accept the COVID-19 vaccine compared to younger HCWs. Similarly, the acceptance was higher among HCWs who were previously infected with SARS-CoV-2, updated themselves on the development of vaccines against COVID-19, perceived COVID-19 to be a severe disease, complied well with COVID-19 preventive guidelines, those who were highly anxious about contracting COVID-19 and those who feared about the side effects of the vaccines (Table 4).

Table 5 indicates significant association between COVID-19 vaccine acceptance and income of the country. Notably, HCWs from UM-HICs had significantly higher intention to be vaccinated compared to those from L-LMICs (75% vs. 62%, *p* < 0.001). This trend was almost dominant across the various demographics of this survey (Table 5).

Logistic regression statistics identified nine predictors of vaccine acceptance among the study population. Greater acceptance can be anticipated among male HCWs, those 50 years of age or older, native HCWs and HCWs from the UM-HICs. Additionally, HCWs who were not previously infected with COVID-19, those who followed the updates about COVID-19 vaccines, those who perceived COVID-19 to be a severe disease, those with higher levels of anxiety about contracting COVID-19 and those who had safety concerns about the vaccines indicated greater intention to be vaccinated against COVID-19 (Table 6).

## Discussion

The success in the fight against COVID-19 rests largely on successful global vaccination coverage. Our study contributes to the literature on potential global COVID-19 vaccine acceptance among HCWs in six L-LMICs and six UM-HICs across four continents. Although 69% of the participants in our study indicated vaccine acceptance, our findings indicate high heterogeneity between the two groups of nations and within each group. Even though, 75% of the participants from UM-HICs indicated their agreement to accept the vaccine, only 62% from L-LMICs agreed to do so. Participants from Malaysia and Brazil, both UM-HICs, indicated the greatest acceptance rates at 98 and 97%, respectively, whereas those from L-LMICs in the African continent, including Egypt, Kenya, Nigeria and Sudan indicated the lowest with 40, 49, 50, and 57% acceptance rates, respectively. Among the L-LMICs, India and Pakistan showed better acceptance rates of 69 and 76%, respectively. Our results indicate an overall acceptance rate of 69%, which could be just enough to attain population immunity. However, the far-from-universal willingness is definitely a cause for concern and stresses the need for implementation of country- and culture-specific strategies.

Currently, there is inadequate information on vaccine acceptance among HCWs in L-LMICs, especially in the African continent. Two studies in the African continent before the start of vaccination campaigns indicated wide ranging acceptance rates of 79 and 40% (15). A study in the Democratic Republic of Congo in the early phase of the pandemic indicated that only 28 percent of the HCWs were willing to accept a vaccine against COVID-19 (12). Our results represent attitudes following the implementation of the vaccination campaigns, indicating low acceptance rates, as reported in other studies (16, 17). Reasons for this could be the lower number of reported infections and deaths in L-LMICs as compared to UM-HICs, mistrust in authorities, lack of access to vaccines, etc. (18).

Although the pace of vaccine roll-out has been slow in the L-LMICs, as of 30 September 2021, more than 60% of the population in all surveyed UM-HICs, except Jordan, has received at least one dose of the vaccine. Interestingly, in our study, among the UM-HICs, only participants from Jordan indicated very low vaccine acceptance rates (53%). The lack of trust in health authorities (only 28%) could be a major reason for this as suggested in other large-scale studies in Jordan indicating similar acceptance rates (19, 20). This could also be the case in L-LMICs in the African continent, where trust in healthcare authorities was low, due to their mishandling of the pandemic and laxity in procurement of vaccines, indicating the need for a bottom-up rather than a top-down approach. Since mistrust in authorities could translate to vaccine hesitancy or even denial, community engagement through local, tribal and religious leaders would be a viable option in L-LMICs. Surprisingly, even though Brazil indicated the lowest trust in health authorities among all the surveyed countries (only 10%), their vaccine acceptance rate was high (97%) as reported in other studies (17, 21, 22). This could be due to the high infection and mortality rate in Brazil throughout the pandemic and an increase during the study period (23).

Even though vaccine inequality in L-LMICs is a major barrier toward attaining population immunity, it could also be a reason for the low acceptance rates (24). Results from our study suggest that a higher percentage of participants from L-LMICs preferred out of fear of vaccines (35%) or consideration for others (49%), to wait for others to get vaccinated before accepting it themselves, as compared to those from UM-HICs (19 and 37%, respectively). This could be an indication of potential future increase in vaccine acceptance in L-LMICs if policymakers can address safety concerns and misinformation effectively and promote trust in health authorities. It is of concern, that some rich countries have begun giving out third doses while several L-LMICs have exhausted the limited vaccines supplied to them through the COVAX facility (25). For example, Rwanda administered 96% of the doses given to it in March within the first 2 weeks, and in June, Nigeria exhausted all of the 3.9 million doses it was given in the first phases of vaccination (26). Currently COVAX has shifted its goal of delivering two billion doses from this year to 2022 (6). Therefore, an impending challenge more serious than vaccine hesitancy in L-LMICs, that will need to be addressed by policymakers is increasing supply to meet a potential increase in demand. However, even if sufficient vaccines are supplied, lack of access or inconvenience in getting vaccinated could also translate to hesitancy. Therefore, strategies aimed at addressing hesitancy must also include making vaccines easily accessible in safe and convenient locations.

After performing logistic regression analysis, we came out with several predictors of global vaccine acceptance in the overall sample, with the income of the country being one of them. Interestingly, bivariate analysis among the two groups of countries also indicate similar significant associations between the intention to vaccinate and the parameters, but with variations in the margins of association. For example, consistent with other studies on HCWs and the general population, we found higher vaccine acceptance among males than in females, with the margin being comparatively lower in L-LMICs (27, 28). This could probably be due to the greater perception of COVID-19 severity, lower belief in conspiracy theories surrounding the disease and the vaccines, and greater access to vaccines among males. The initially reported greater risk of blood clots with certain vaccines in females and their safety concerns in pregnant ladies could also have created a negative attitude toward vaccines among them (28–30). Apart from educating females on the safety of the vaccines, policymakers should pay particular attention to providing them easy access to vaccines, especially in L-LMICs. Higher age was shown to be directly proportional to vaccine acceptance, with people over 50 years of age being most likely to accept the vaccine. Numerous studies on a global scale have reported a similar trend (31). The reason could again be the greater probability of disease severity in older individuals. On a similar note, individuals who had one or more comorbidities were more likely to accept a vaccine than those who didn't, probably due to their fear of a greater likelihood of the disease to advance to critical stage or even death.

An interesting finding in our study is the strong association between the intention to accept the vaccine and participants who updated themselves on its development. This underlines the importance of incorporating wide ranging educative strategies, on the development, safety profile and efficiency of the vaccines, through social media, public signboards and by involving respected community figures. As suggested in numerous studies, the strongest predictor of vaccine acceptance in our study is the concerns on the safety profile and side effects from the vaccines (11, 18, 32). Since our study was conducted at a time when reports of specific vaccine related adverse events, even though rare, were being widely reported in social and mass media, we find this association logical.

An emerging threat to vaccine acceptance and attaining population immunity is the increasing reports of COVID-19 vaccine breakthrough infections in fully vaccinated people (33). Although a majority of them are asymptomatic or mild, they could cause hesitant individuals to deny vaccines altogether. Another stumbling block is the provision of third vaccine doses in HICs, which could widen vaccine disparity, thereby jeopardizing vulnerable populations in L-LMICs. Although the WHO has called for a moratorium on countries giving out third doses, several countries, including the USA, have disregarded that call (34). These concerns should be appropriately addressed by the concerned authorities.

Although global vaccine distribution has skewed heavily toward higher-income countries, the modest levels of vaccine acceptance we identify in L-LMICs suggest that apart from educating the public on the necessity to accept vaccines, prioritizing distribution to them may be an efficient way to achieve immunity on a global scale and prevent novel variants from emerging. Vaccine hesitancy is one dilemma affecting most countries, if not all. However, L-LMICs are facing additional hurdles like lack of access to vaccines. Since there is a possibility that the vaccine acceptance rates may increase if access to vaccines is increased, especially in L-LMICs, governments of L-LMICs, policymakers, donor nations and NGOs like the WHO and COVAX should use every opportunity to increase supply to L-LMICs to meet the needs of their populations. The concerned authorities should work inclusively with governments of L-LMICs to focus on converting positive intentions into uptake by also investing in local supply chains and delivery. Educating health workers and community leaders and engaging them to deliver information and messaging focused on vaccine effectiveness and safety will go a long way in addressing vaccine hesitancy.

Our study has a few limitations. Since there could be a number of HCWs, especially in L-LMICs who would be willing to get vaccinated but do not have access to a COVID-19 vaccine, there is a possibility of bias. Our results represent the attitudes of HCWs at a particular point in time and is expected to change as the vaccination campaigns gather pace, depending on several factors, including rate of disease transmission and mortality, first-hand information from vaccinated friends and relatives, change in perception of disease severity and trust in authorities, etc. (35, 36). Therefore, more studies are necessary to collect snapshots at different points in time to capture changes in mass behavior and prioritize vulnerable populations accordingly. Such studies will be useful during possible future outbreaks as well. During our study period there were several reports of vaccine related serious adverse events forcing governments to put on hold, opt out or halt the use of specific vaccines (37). However, the decline in the reported adverse events and the assurances by concerned authorities have helped in gaining back public trust and combating vaccine hesitancy. Another limitation is that our results are not representative of the population at large, as we used convenience sampling. However, they do serve as a guide to policymakers and stakeholders. Yet another limitation is the mode of study. Since we used a web-based self-administration mode of survey, there could be potential bias among the participants in responding to the survey questions. However, due to the restrictions related to the pandemic, this was the best mode currently available.

## Data Availability Statement

The questionnaire used in the current study is not publicly available due to certain restrictions. However, it is available from the corresponding author (Mohammed Noushad) on reasonable request.

## Ethics Statement

This study was conducted according to the guidelines of the Declaration of Helsinki and approved by the Research Committee of College of Dentistry, Dar Al Uloom University, Saudi Arabia (COD/IRB/2020/2). The participants provided their consent to participate in this study.

## Author Contributions

MN and MZN: conceptualization, methodology, and writing—original draft. MN, SR, MZN, IA-S, MH, AY, MA, AK, PK, FN, AE, MD, ASA, SA, NA, GA, AS, ABA, AD, RM, NL, AHu, AHa, FS, HO, MA-A, MRD, LS, AB, and AA: investigation and data collection and writing—review for important intellectual content and editing. MN, MZN, and SR: data analysis. All authors contributed to the article and approved the submitted version.

## Conflict of Interest

Author AE is employed by Hamad Medical Corporation. The remaining authors declare that the research was conducted in the absence of any commercial or financial relationships that could be construed as a potential conflict of interest.

## Publisher's Note

All claims expressed in this article are solely those of the authors and do not necessarily represent those of their affiliated organizations, or those of the publisher, the editors and the reviewers. Any product that may be evaluated in this article, or claim that may be made by its manufacturer, is not guaranteed or endorsed by the publisher.
